# *In vitro* and *in vivo* antimicrobial potential of lithium complex against multi-drug resistant *Acinetobacter baumannii*

**DOI:** 10.1128/spectrum.01930-23

**Published:** 2023-10-20

**Authors:** Moatter Zehra, Yamina Usmani, Jazib Shafiq, Ajmal Khan, Muneeza Zafar, Munazza Raza Mirza, Syed Raza Shah, Ahmed Al-Harrasi, Syed Mehmood Hasan, Amber Farooqui, Ayaz Ahmed

**Affiliations:** 1 Dr. Panjwani Center for Molecular Medicine and Drug Research, International Center for Chemical and Biological Sciences, University of Karachi, Karachi, Pakistan; 2 H.E.J. Research Institute of Chemistry, International Center for Chemical and Biological Sciences, University of Karachi, Karachi, Pakistan; 3 Natural and Medical Science Research Center, University of Nizwa, Birkat Almouz, Oman; 4 Department of Pathology, Jinnah Sindh Medical University, Karachi, Pakistan; 5 Translational Medicine Program, The Peter Gilgan Centre for Research and Learning, The Hospital for Sick Children, Toronto, Ontario, Canada; The University of North Carolina at Chapel Hill, Chapel Hill, North Carolina, USA

**Keywords:** lithium complex, *Acinetobacter baumannii*, biofilm, multi drug resistant, nosocomial infections, metal complexes

## Abstract

*Acinetobacter baumannii* is a Gram-negative pathogen, accountable for lethal nosocomial infections, owing to high pathogenicity and multi-drug resistance (MDR). Hence, the World Health Organization has prioritized this bacterium against which new pharmacophores are required urgently. Therefore, this study evaluates the antimicrobial potential of lithium complex based on salicylic acid, and 1,10-phenanthroline {[Li(phen)_2_sal]} against MDR *A. baumannii* (MDRAB) through growth inhibition, growth curve assay, biofilm inhibition, and eradication alongside microscopy. The mechanism of action was proposed by flow cytometry and proteomic analysis. Moreover, *in vivo* efficacy was assessed using an *A. baumannii*-induced pneumonia model in mice. Results showed that lithium complex significantly inhibited the bacterial growth at 16–32 µg/mL. Growth kinetics revealed that it retarded the bacterial growth after 2–3 h of treatment. Lithium complex significantly inhibited and reduced >70% of biofilms at the MIC level which is a key problem of indwelling and prosthetic devices. Light and atomic force microscopy further indicated disarticulated biofilm and ruptured cellular membrane post-lithium complex treatment. Proteomic analysis revealed that the compound exerted oxidative stress on *A. baumannii* which may contribute to cell rupturing. Data are available via ProteomeXchange with identifier PXD044293. Flow cytometry confirmed its role in cytoplasmic membrane disintegration. The lithium complex ameliorated the pneumonia symptoms at non-toxic doses of 20 and 40 mg/kg in the pre-clinical *A. baumannii*-induced pneumonia model as evident by the significant reduction in bacterial burden and parenchymal inflammation. Taken together, these findings suggest that the lithium complex can be a potential drug candidate against MDRAB.

**IMPORTANCE:**

Multi-drug resistance (MDR) by virtue of evolving resistance and virulence mechanisms among *A. baumannii* is a global concern which is responsible for lethal hospital-acquired infections. Therefore, it is crucial to develop new therapeutics against it. Metal complexes are compact structures with diverse mechanisms that the pathogens cannot evade easily which make them a strong drug candidate. In this study, we assessed the *in vitro* and *in vivo* efficacy of lithium complex {[Li(phen)_2_ sal]} against biofilm-forming MDR *A. baumannii*. The lithium complex displayed strong antimicrobial activity and reduced the pre-formed mature biofilm which is key barrier for antimicrobial action. Moreover, it employs oxidative stress as one of its mode of actions and causes cellular rupturing. Lithium complex was non-toxic and was significantly effective to overcome pneumonia in mice model. These results highlight the untapped potential of metal complexes that can be explored and utilized for combating notorious *A. baumannii* infections.

## INTRODUCTION

ESKAPE pathogens correspond to a group of opportunistic and drug-resistant pathogens that have emerged as a cause of various life-threatening hospital-acquired infections (1). ESKAPE pathogens include *Enterococcus faecium, Staphylococcus aureus, Klebsiella pneumoniae, Acinetobacter baumannii, Pseudomonas aeruginosa*, and *Enterobacter* species (2). In recent years, injudicious and persistent use of antibiotics has caused these pathogens to acquire multiple drug resistance. Consequently, ESKAPE pathogens are estimated to cause up to 10 million deaths by 2050 (3). This group of pathogens has presented a threatening complication owing to its pattern of resistance and transmission resulting in illnesses with prolonged hospital stays with heavy financial burden on the global economy (4).

*Acinetobacter baumannii* is a coccobacillus and a Gram-negative member of ESKAPE pathogen that is known to cause infections in critically ill patients at intensive care units (ICUs). It contributes to around 2%–10% of hospital-acquired infections (5) and has the propensity to develop a variety of resistance mechanisms that can render the currently available treatments ineffective. These mechanisms include drug modification, target modification through genetic mutation, increased efflux pump activity, and altered metabolic pathways (6).

Biofilm formation is one of the resistance mechanisms employed by *Acinetobacter baumannii* to circumnavigate the antibiotics by preventing drug penetration (7). It is an assemblage of intercommunicating bacterial population that is entangled in extracellular polysaccharides, DNA, and proteins. Eventually, it matures and develops into an enclosed and protective consortium that can adhere to biotic and abiotic surfaces to prolong the survival of bacteria (8). Various studies have reported that *Acinetobacter baumannii* has a higher biofilm formation rate than other species which has contributed significantly to its pathogenesis to cause persistent infections that are challenging to eradicate (9). These hospital- and community-acquired infections include catheter-associated urinary tract infections, ventilator-associated pneumonia, bacteremia, and sepsis (10).

There are various classes of antibiotics including fluoroquinolones, aminoglycosides, carbapenems, and beta-lactams that have been traditionally used in the treatment of *Acinetobacter baumannii*-related infections. However, it has been reported that 45% of the strains isolated in clinical settings are multi-drug resistant (11, 12). Hence, with the emergence of carbapenem-resistant strains, colistin has been used as a last resort drug, but unfortunately, isolation of colistin-resistant strains is also being reported recently (13 – 15). This alarming situation led the World Health Organization and the Center of Disease Control (CDC) to declare *A. baumannii* as a critical pathogen in 2017, against which the development of new pharmacophores is imperative (16, 17). Ironically, most of the drugs under development are derivatives of the drugs already in the market which increases the chances of development of bacterial resistance against them (18). Hence, exploration for other alternatives has become inevitable.

In recent years, various metal-based compounds have been tested for anti-cancer activity, malaria, and neurodegenerative diseases but little has been explored regarding their antibacterial activity. Several studies have suggested that metallodrugs can be used alone or in combination due to their access to unique and various modes of action that can make them less prone to bacterial resistance mechanisms (19, 20). These novel and nonspecific mechanisms of action include reactive oxygen species (ROS) production, ligand exchange, and bioactive molecules production (21). As compared to the organic compounds, they have a higher three-dimensional structure and can access different geometries which play a major role in their high clinical success and bioactivity (22).

It is mainly due to the potential toxic effects that only a few metals like silver and bismuth have been developed into metallodrugs against bacterial infections (23, 24). However, with the rise of antimicrobial resistance all around the globe, alkali metal-based compounds have resurfaced as potential drug candidates (25). Among these alkali metals, lithium-based compounds are recently being highlighted for their antibacterial potential (26, 27). Hence, in this study, lithium complex based on salicylic acid and 1,10-phenanthroline has been explored for its potency against multi-drug-resistant *A. baumannii* (MDRAB) through different experimental approaches. Its synthesis and structure have already been reported previously (28). As little has been explored regarding the potential mechanism of action against Gram-negative pathogens, proteomic analysis has been employed to decipher it against *A. baumannii*. Moreover, as only a few metallodrugs have been tested *in vivo,* efficacy of lithium complex was also assessed in *A. baumannii*-induced pneumonia model.

## MATERIALS AND METHODS

### Bacterial strains and media

*A. baumannii* (ATCC 19606) and its clinical isolates from various diagnostic laboratories were cultured on heart infusion broth (Oxoid, UK) and MacConkey agar (Oxoid, UK). The clinical strains were isolated from the endotracheal aspirate of infected individuals. All bacterial strains were maintained on tryptic soy agar/broth (Oxoid, UK). For all the experiments, bacterial strains were aerobically incubated at 37°C for 24 h.

### Phenotypic and genotypic characterization of *A. baumannii* clinical strains

All the clinical strains of *A. baumannii* were then identified using Gram staining and 16S rRNA-specific primers. The resistance profile of strains was evaluated using breakpoints of different antibiotics through microbroth dilution assay as recommended by Clinical and Laboratory Standards Institute (29). Only MDR and strong biofilm-forming strains of *A. baumannii* were selected for further experiments.

### Growth inhibition assay

Microbroth dilution assay was used for assessing MIC of lithium complex against *A. baumannii* and its clinical strains (29). Briefly, the compound was twofold serially diluted in a 96-well plate (NEST Biotechnology, USA) with media and was inoculated with bacterial inoculum (5 × 10^5^ CFU/mL). The plate was incubated at 37°C for 24 h. Next day, 10% (vol/vol) resazurin dye (7-hydroxy-3H-phenoxazin-3-one-10-oxide) was added in each well and was re-incubated for a further 2 h. The plate was observed for the reduction of resazurin dye to determine MIC visually. Then, MIC_90_ was determined by calculating percentage growth inhibition, from the absorbance recorded at 570 nm and 600 nm through spectrophotometer (Thermo Scientific, USA) (30).

### Growth curve assay

Growth kinetics of all the *A. baumannii* strains treated with lithium complex was evaluated using growth curve assay. For this, lithium complex was prepared at various concentrations of 0.5× MIC, MIC, and 2× MIC in a 96-well plate and inoculum (5 × 10^5^ CFU/mL) was added. Positive (media with bacterial culture) and negative controls (only media) were also added into the plate. The plate was incubated at 37°C and optical density (OD) was measured at different time points (i.e., 0, 1, 2, 3, 4, 5, 6, and 24 h). The growth inhibition curve was obtained by plotting time against OD.

### Biofilm inhibition assay

The biofilm inhibitory potential of lithium complex at MIC dose was evaluated by crystal violet assay as described earlier (31). Plate was prepared as mentioned in antimicrobial assay and after 24 h of incubation, the plate was washed with sterile water, and heat was fixed at 65°C for 40 min. Then, it was stained with 0.1% (wt/vol) crystal violet followed by washing to remove excess dye. To solubilize the dye, 30% (vol/vol) glacial acetic acid was added, and absorbance was measured at 590 nm.

### Biofilm eradication assay

Biofilm eradication assay was performed to ascertain the biofilm eradication potential using crystal violet dye. Static biofilm was grown by inoculating 5 × 10^5^ bacterial cells in fresh media and they were allowed to form biofilm for 24 h. Later, sterile phosphate buffer saline (PBS) was used to remove non-adherent bacterial cells. Fresh media containing 16 µg/mL lithium complex was added in well-containing pre-formed biofilm and plate was further incubated for 24 h at 37°C. Next day, the plate was washed and stained with crystal violet as mentioned above to determine OD and percent biofilm eradication.

### Biofilm viability assay

Resazurin dye was used to determine the biofilm viability at MIC level (32). Plate was prepared as mentioned in biofilm eradication assay. After the overnight incubation with the compound, resazurin was added at 10% (vol/vol) to the plate, followed by 2-h incubation. Later, absorbance was measured at 570 nm and 600 nm to calculate growth inhibition.

### Microscopic evaluation

Light microscopy (LM) and atomic force microscopy (AFM) were used to visualize the effect of lithium complex on biofilm eradication and morphological transformation. For LM, plate was prepared and processed as described earlier for biofilm eradication assay. The plate was visualized under 20× bright field objective lens using TE2000-E microscope (Nikon) at microscope imaging facility at the International Center of Chemical and Biological Sciences, University of Karachi. For AFM, bacterial biofilm was grown on sterile silicon chips and treated with lithium complex at MIC for 24 h. Next day, silicon chips were washed with sterile distilled water to remove non-adherent cells, followed by heat fixation of bacterial biofilm for 15–20 min. Images were captured under acoustic atomic force microscopy (ACFAM) mode with force constant of 42 Nm^−1^ and resonance frequency of 330 kHz.

### Whole proteins extraction and proteomic analysis via mass spectrometry

*A. baumannii* ATCC 19606 was cultured in 100 mL tryptic soy broth (TSB) at 37°C under constant shaking until the OD reached 0.5. For the treated sample, the culture was further incubated with the compound for 2 h. Later, the bacterial cells were washed with normal saline and pelleted by centrifugation at 3,500 rpm for 15 min at 4°C. Both the treated and untreated samples were then suspended in a 3 mL disintegration buffer, as reported previously (33). The samples were then sonicated on ice [3 × 5 min (0.2 duty cycles), 30 W amplitude], and unbroken cells were separated by centrifugation at 3,500 rpm for 5 min. Supernatant was collected and centrifuged at 3,500 rpm for 30 min to fully isolate the proteins in buffer. Samples were then passed through 0.45 µm syringe filter (Millipore, USA) and stored at −20°C. Samples were quantified through Qubit Protein Assay Kit (ThermoFisher Scientific, USA).

Initially, 1 M ammonium bicarbonate (160 µL) was added to protein samples (200 µg) to adjust the basic pH of the samples. Then, 45 mM dithiothreitol was added and the mixture was incubated at 95°C for 30 min on thermomixer (Eppendorf AG, Germany). Samples were then allowed to cool down, and in the next step, 100 mM iodoacetamide was added for alkylation. Samples were then incubated in the dark for 15 min at room temperature. Afterward, the volume of samples was adjusted by adding 700 µL of distilled water. Then, trypsin was added at 1:50 ratio and samples were incubated overnight at 37°C in shaking incubator (34, 35). Trypsin activity was stopped by adding 50 µL of 1% (vol/vol) trifluoroacetic acid (TFA). Later, samples were desalted through in-house-assembled reverse phased C18 cartridges, and the eluents were vacuum dried completely. Samples were dissolved in 0.1% (vol/vol) TFA and were quantified by using Qubit kit. Samples were then run in triplicate on nano-liquid chromatography tandem mass spectrometry(LC–MS/MS) on an Orbitrap Q-Exactive HF-X (ThermoFisher Scientific, USA) with an EASY-LC 1000 system (ThermoFisher Scientific USA) (36).

The mass spectrometry proteomics data have been deposited to the ProteomeXchange Consortium via the PRIDE (37) partner repository with the data set identifier PXD044293. All the raw files generated by MS were transferred to MaxQuant (v2.3.2, Matrix Science, UK) for protein identification and to obtain label-free quantification (LFQ) intensities for quantification. The search was performed through Andromeda with default search settings at 1% discovery rate. The generated spectra were then matched against the *Acinetobacter baumannii* reference fasta file from UniProt (https://www.uniprot.org/) database. Search parameters were adjusted as carbamidomethylation was set as fixed modification, and methionine oxidation was set as variable modification alongside protein amino termini acetylation (38). Proteins were identified based on one unique peptide and quantified based on two unique peptides. False discovery rate (FDR) cutoff was set to 5%. For data analysis, Perseus software (v2.0.6.0) was used. The Student’s *t*-test was applied to find the significant difference in LFQ intensities among groups. Moreover, differential proteins were then analyzed for Gene Ontology (GO) through STRING database (version 11.5) (https://string-db.org/).

### Membrane potential and integrity assay

The rapid action of lithium complex on membrane potential and integrity of *A. baumannii* was evaluated through DiBAC_4_ [bis-(1,3-dibutylbarbituric acid) trimethine oxonol]. Initially, 5×10^5^ CFU/mL cells were incubated in 1 mL media for 24 h at 37°C. After centrifugation of cells at 5,000 *g* for 5 min, pellet was resuspended in sterile PBS. The lithium complex at MIC dose was added to their respective tubes and then incubated for 2 h at 37°C. After that, DiBAC_4_ at 5 µg/mL was added in treated and non-treated cells and again incubated for 30 min. Excess dye was removed by washing through centrifugation and pellet was resuspended in 1 mL of sterile PBS. Then, samples were analyzed by flow cytometry through FACS Celesta (FACS BD Diva TM software).

### *In vivo* efficacy in mice pneumonia model

Pneumonia model in mice was developed as previously reported with slight modifications (39). Mice were divided into five groups (*n* = 10) such that the first group was kept as infected control, three groups were administered compound at various concentrations (10, 20, and 40 mg/kg), and the fifth group was administered normal saline only. Briefly, cyclophosphamide-induced neutropenic BALB/c mice were inoculated with 50 µL (110^8^ CFU) of *A. baumannii* clinical strain 1. Treatment with lithium complex at 10, 20, and 40 mg/kg was started 4 h post inoculation. The drug was administered intraperitoneally twice per day in each respective group. The fifth group received normal saline and served as the vehicle control as well. All the mice of each group were observed for clinical symptoms and five of them were euthanized at 24 h and 48 h to collect lungs for bacterial load and histopathology (40). Furthermore, kidney and liver were also collected for histopathology to assess toxicity at the highest dose of 40 mg/kg. For bacterial count, lungs were homogenized in PBS, serially diluted (10-fold), and plated on trypic soy agar (TSA) plates. Lungs for histopathology were fixed in 10% formalin, processed in graded alcohol, xylene, and embedded in paraffin to make a block. Tissues were then sectioned (5 µm) and stained in hemoxylin/eosin dye for microscopy. Histopathological analysis was performed by an independent histopathologist who analyzed the samples for inflammation, necrosis, and fibrosis as described earlier (41).

### Statistical analysis

All assays were performed in triplicate. Results were represented as mean ± standard deviation. One-way analysis of variance (ANOVA) was used to analyze whether lithium complex presence is significantly different compared to control levels. Bacteriology data in *in vivo* studies were assessed using two-way ANOVA followed by Sidak’s multiple comparison test. Data were statistically computed using GraphPad Prism 8.1.1. such that *P* < 0.05 was considered significant.

## RESULTS

### Clinical strains of *Acinetobacter baumannii*

Fifteen clinical isolates were identified as *A. baumannii* through colony morphology, and Gram staining. The isolates were further confirmed through PCR by 16S rRNA-specific primer for *A. baumannii*. Three of the clinical strains (CS) were found to be resistant against meropenem, gentamicin, ciprofloxacin, ceftriaxone, and colistin and had strong biofilm formation. Hence, MDRAB was selected to assess antibacterial efficacy of lithium complex.

### Antibacterial efficacy of lithium complex against *A. baumannii* strains

**Fig 1:**
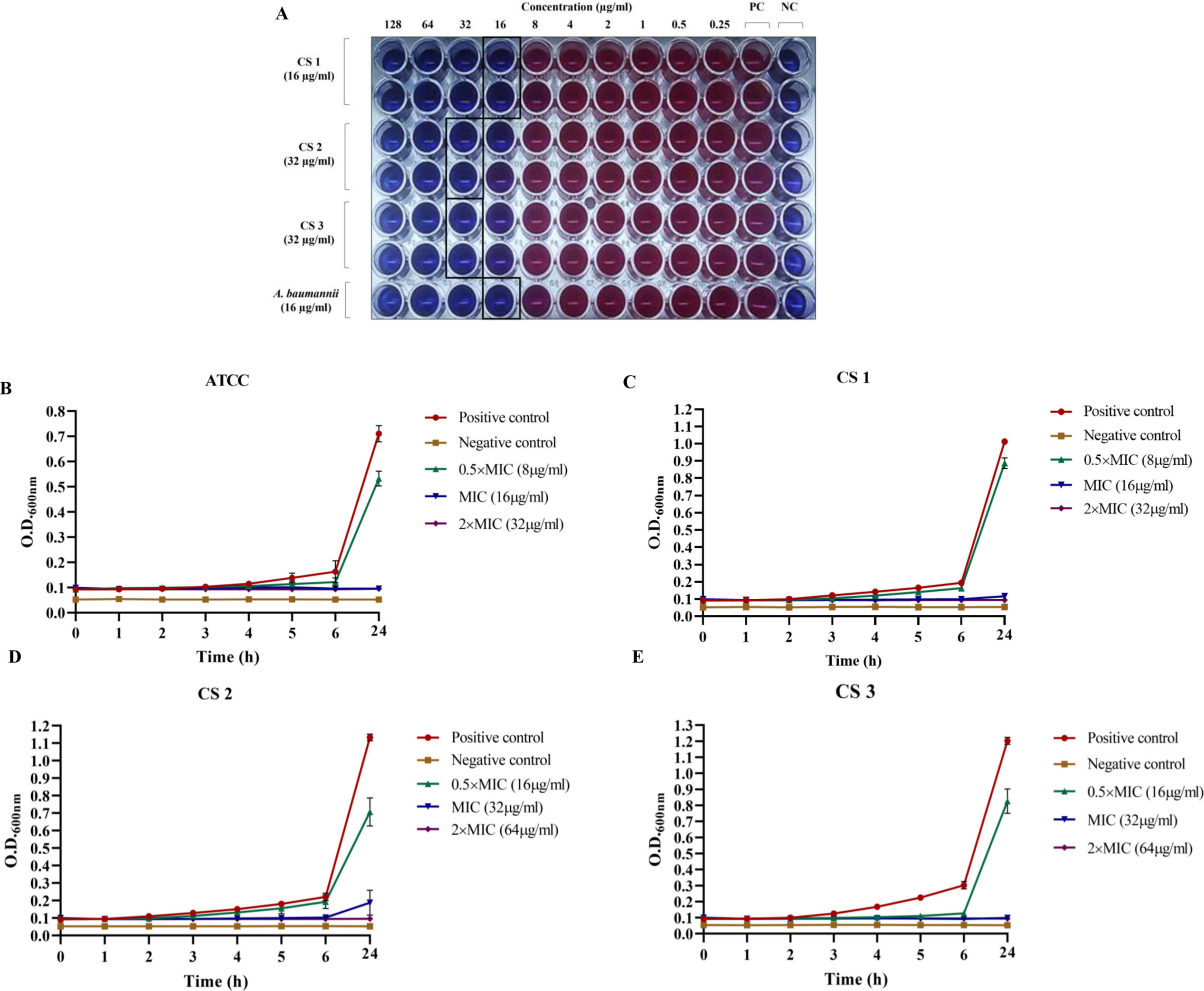
(**A**) Growth inhibition by lithium complex. Susceptibility of ATCC and clinical strains of *A. baumannii* against lithium complex. Selected wells represent the MIC of test compound alongside positive controls (PCs) and negative controls (NCs). (**B–E**) Growth curves of lithium complex treated *A. baumannii* ATCC 19606 and clinical strains (CS 1, CS 2, and CS 3). PC is media with inoculum while NC had media only.

**Fig 2:**
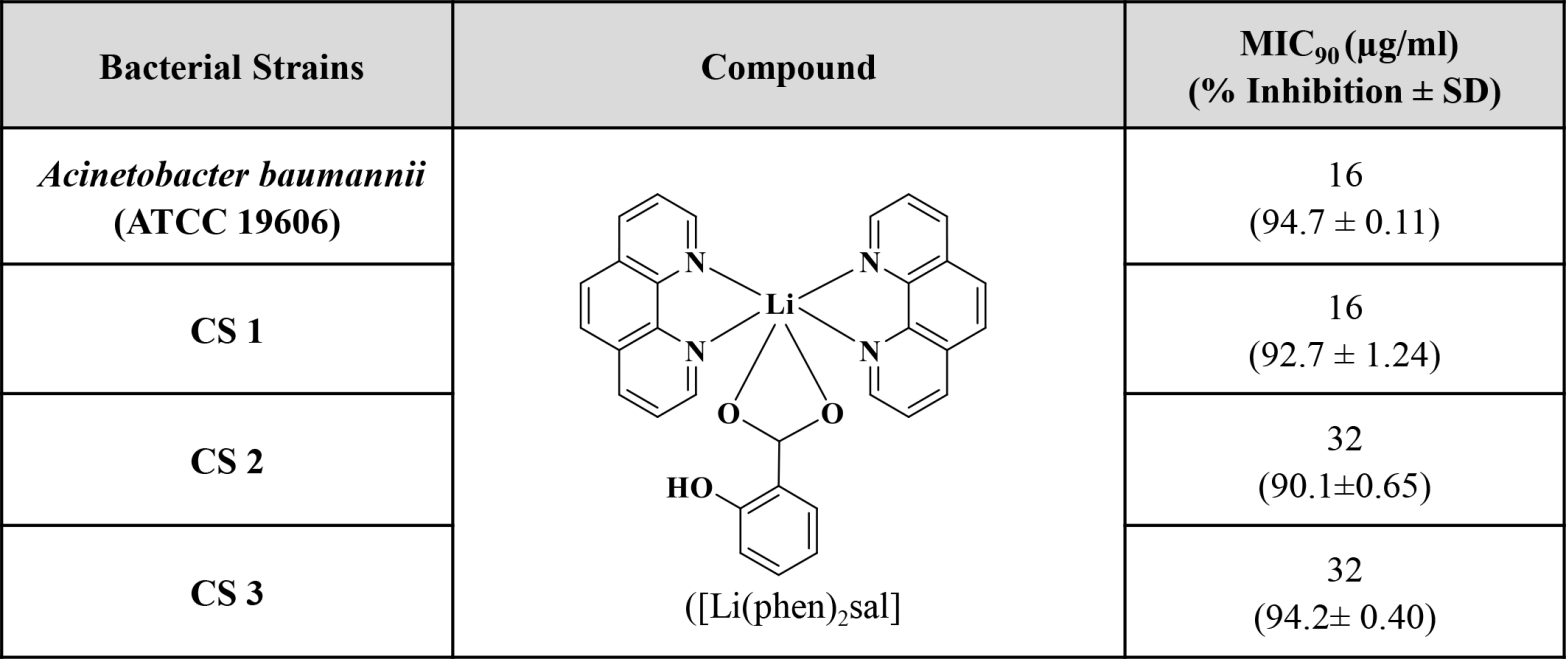
Antibacterial activity of lithium complex (µg/mL).

The lithium complex showed potent antimicrobial activity against *A. baumannii* strains. The MIC of *A. baumannii* and clinical strain 1 (CS 1) was 16 µg/mL with >90% growth inhibition while the other two clinical strains had MIC of 32 µg/mL, respectively, and inhibited >87% of bacteria (Fig. 1A and 2).

### Growth kinetics of *A. baumannii* at MIC and sub-MIC of lithium complex

Growth kinetics of *A. baumannii* treated with different concentrations (0.5× MIC, MIC, and 2× MIC) of lithium complex was analyzed at various time points. It was observed that compound began to show inhibition against *A. baumannii* strains after 2–3 h at MIC and 2× MIC doses. The growth inhibition of ATCC strain started after 3 h such that no growth was observed until 6 h of incubation (Fig. 1B). In contrast, in CS 1, significant inhibitory effect was observed after 3 h, while CS 2 and CS 3 were significantly inhibited after 4 h (Fig. 1C through E). At 24 h, inhibitory effect of MIC dose was more than the positive control in all *A. baumannii* strains. Its inhibitory potential was alike at 2× MIC against all the strains except in CS 2 where efficacy of 2× MIC was slightly high. The dose of 0.5× MIC showed lower efficacy as compared to the MIC and 2× MIC dose in all the *A. baumannii* strains. As the compound showed similar potency at MIC level against all *A. baumannii* clinical strains, ATCC culture and CS 1 were selected for further experiments.

### Lithium complex inhibition and reduction of pre-formed biofilm

**Fig 3:**
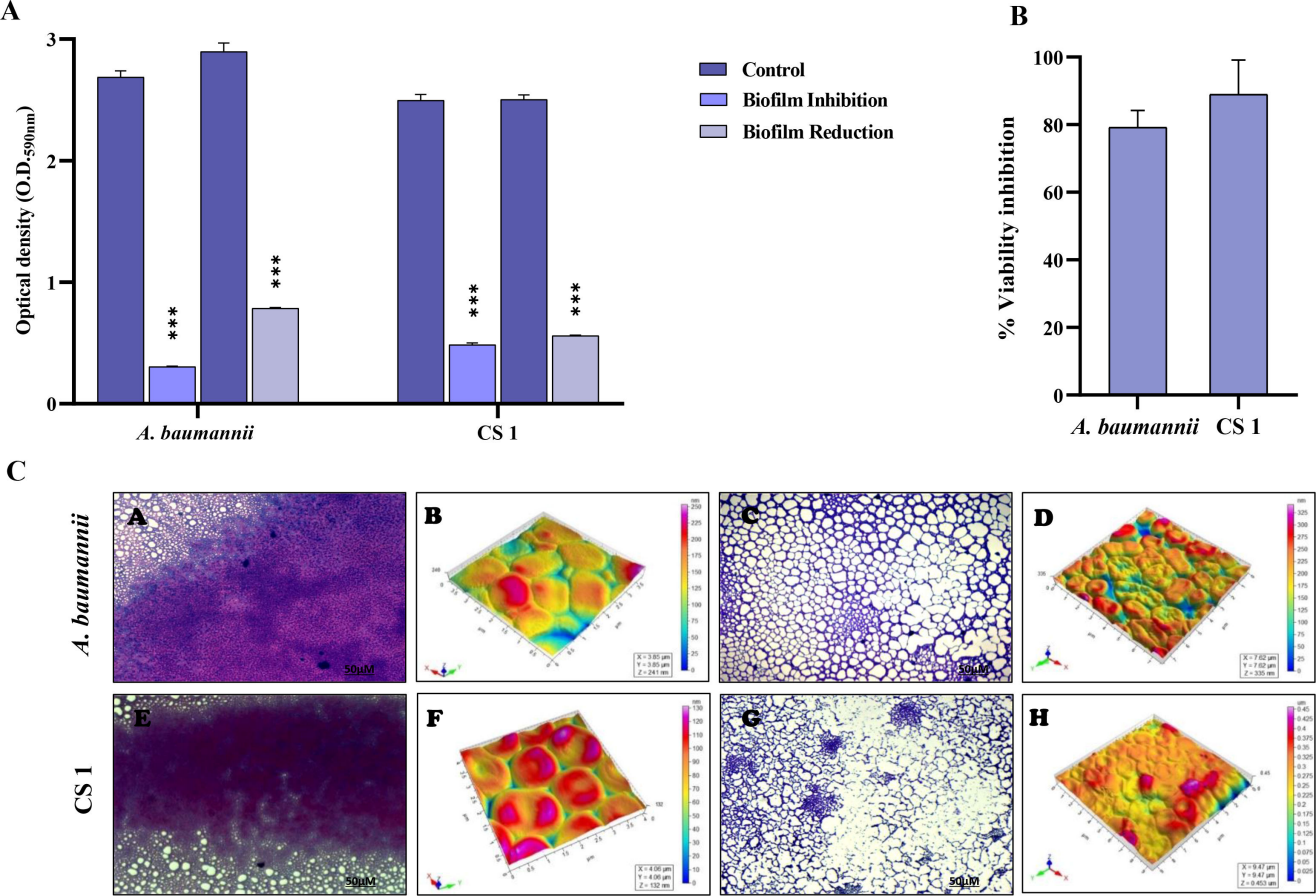
(**A**) Effect of lithium complex on inhibition and reduction of 24-h mature biofilm of *Acinetobacter baumannii* and CS 1 at MICs. The statistical significance was evaluated by comparing each strain with its non-treated control, where *** represents *P* < 0.001. (**B**) Biofilm viability inhibition of *A. baumannii* ATCC and CS 1 after lithium complex treatment. (**C**) LM and AFM images of biofilm after treatment with lithium complex. (**A, E**) and (**B, F**) are the positive control of *A. baumannii* strain, whereas (**C, G**) and (**D, H**) are the biofilms after exposure of lithium complex at MIC.

The lithium complex was further studied for its antibiofilm efficacy against all strong biofilm-forming strains of *A. baumannii*. The compound constrained the biofilm formation of ATCC and clinical isolates in a significant manner as compared to non-treated control (Fig. 3A). It strongly reduced 86%–89% of biofilm at 16 µg/mL against *A. baumannii* and CS 1 after 24 h. After showing effective biofilm inhibition activity, the lithium complex was further investigated for its biofilm eradication ability. Pre-formed 24-h static biofilm of all the *A. baumannii* strains was treated with lithium complex at MIC and it was observed that it significantly exterminates 72%–78% of biofilm against all *A. baumannii* strains at their respective MICs. The lithium complex at inhibitory concentrations significantly reduced the cell viability and metabolic activity of both *A. baumannii* strains within biofilm, i.e., reduction of 79%–89% cell viability (Fig. 3B).

### Lithium complex effect on *A. baumannii* cell morphology in pre-formed biofilm

Visualization and confirmation of pre-formed biofilm reducing potential of lithium complex were further examined through LM. A static biofilm of *A. baumannii* ATCC and CS 1 was treated with lithium complex at respective MICs. The LM images revealed that mature biofilm treated with lithium complex MIC showed large void spaces as compared to compact and dense biofilm in control of these strains (Fig. 3C). Furthermore, AFM was used to assess morphological transformation of bacterial cells within biofilm after treatment with lithium complex for 24 h. It was observed that bacterial cells within the biofilm got detached and lost their morphological structure and integrity compared to the respective controls which exhibited healthy morphology of bacterial cells within biofilm.

### Proteomic analysis revealed lithium complex exerts oxidative stress on *A. baumannii*

**Fig 4:**
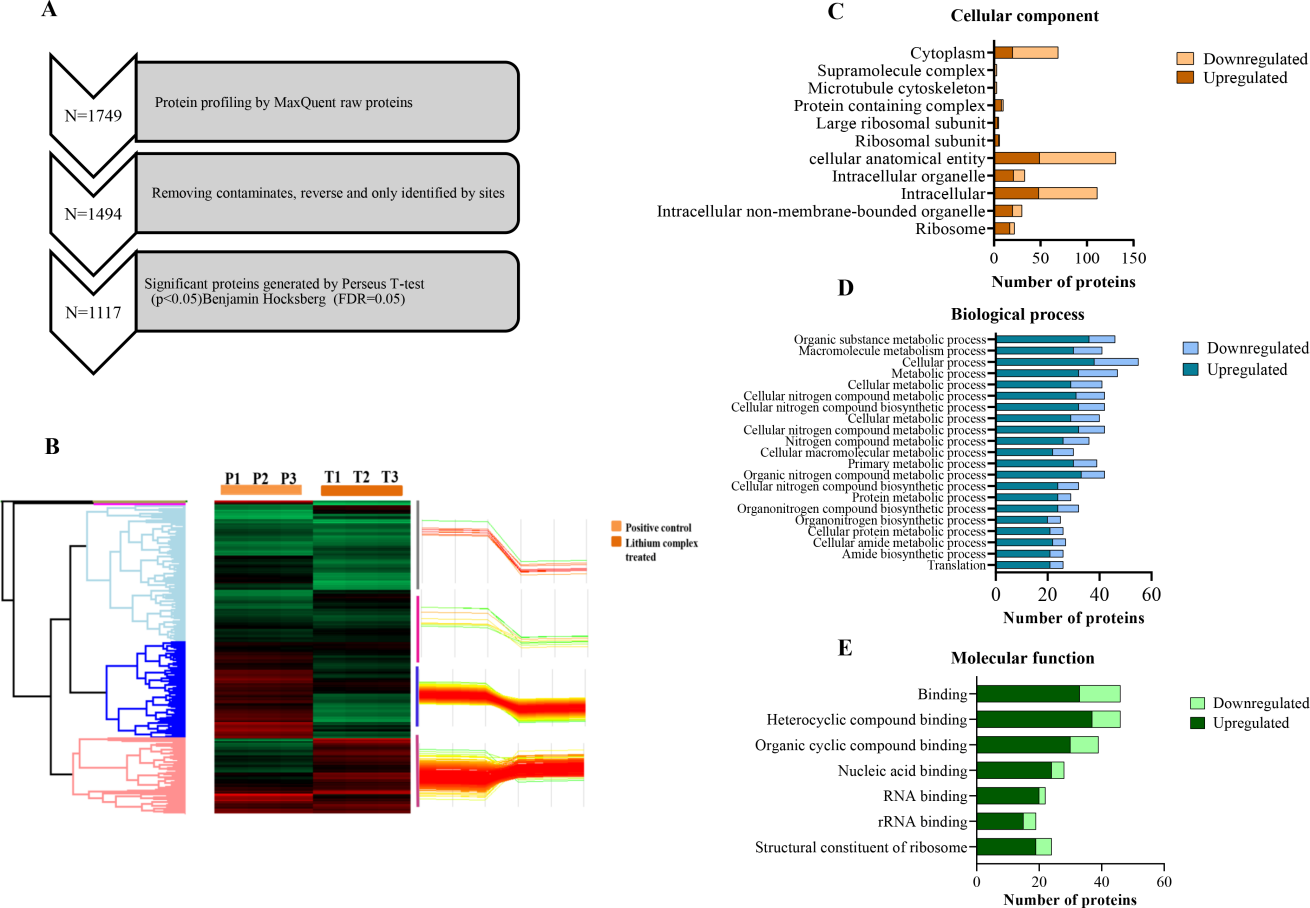
Proteome analysis. (**A**) Each step represents the filters applied and acquired number of proteins. (**B**) Hierarchical clustering and heatmap representation of significantly expressed proteins in positive control triplicate samples (**P1, P2, P3**) and Li complex-treated triplicate samples (**T1, T2, T3**) of *A. baumannii*. (C–E) Functional analysis of significant proteins using GO where (**C**) is biological processes, (**D**) is molecular functions, and (**E**) is cellular components. Each bar represents the total number of proteins characterized into upregulated and downregulated proteins.

Initially, a total of 1,749 proteins were identified by MaxQuant (Fig. 4A). From these, 1,494 proteins were shortlisted after applying filtering steps including removing contaminates, reversed, and only identified by site in Perseus. Significant differences in LFQ intensities of proteins were calculated by *t*-test (*P* < 0.05) resulting in 1,117 proteins. In the next step, hierarchical cluster analysis of the significantly altered proteins was performed to visualize the major changes in the expression of proteins in the lithium complex-treated samples as compared to the positive control. (Fig. 4B). In total, 474 proteins were found to be upregulated and 643 proteins were downregulated. GO based on molecular function, biological process, and cellular components was performed. The lithium complex-treated *A. baumannii* had abundant cytoplasmic, cellular anatomical entities and intracellular proteins (Fig. 4C). Furthermore, there was a differential regulation of cellular and metabolic proteins which are involved in translation, organic substance metabolic process, and macromolecule metabolic processes (Fig. 4D). According to molecular functional analysis, regulation of various heterocyclic compounds, rRNA, and RNA binding proteins were mostly affected (Fig. 4E). Table 1 shows 17 of the significantly altered proteins based on higher fold change values (<1 or >1). These proteins included enzymes involved in oxidative stress, translation, biochemical processes, outer membrane synthesis proteins, and virulence factors. Universal stress proteins, glutathione peroxidase, alkyl hydroperoxide reductase C, and aconitate hydratase were among the most upregulated proteins which are crucial in maintaining the redox balance in *A. baumannii* in response to oxidative stress and are involved in preventing damage at molecular level. Moreover, the most downregulated proteins included enzymes that were involved in biosynthetic processes, proteins crucial for outer membrane synthesis, and virulence proteins that aid bacterial survival in harsh environments.

**TABLE 1:** Significant (*P* < 0.05) protein expression of *A. baumannii* (ATCC 19606) treated with the lithium complex

Protein ID	Protein name	Fold change	*P*-value
Upregulated proteins			
V5VE02_ACIBA	Universal stress protein	1.34	3.311 × 10^–7^
A0A5N0FXK9_ACIBA	Glutathione peroxidase	1.22	1.047 × 10^–6^
V5VA41_ACIBA	Alkyl hydroperoxide reductase C	1.17	3.548 × 10^–6^
V5VDZ0_ACIBA	RecA protein	1.20	1.949 × 10^–6^
V5VBJ4_ACIBA	Aconitate hydratase	1.14	4.446 × 10^–6^
A0A5N0G2V1_ACIBA	Elongation factor Ts	1.40	3.981 × 10^–2^
A0A081H5Y7_ACIBA	Elongation factor Tu	1.36	1.862 × 10^–7^
Downregulated proteins			
A0A5N0G0T0_ACIBA	AraC family transcriptional regulator	0.77	8.317 × 10^–3^
V5V9B2_ACIBA	Membrane protein (OmpA family protein)	0.84	2.172 × 10^–6^
A0A5N0G0T3_ACIBA	Cell envelope integrity protein	0.85	1.120 × 10^–2^
V5VDC0_ACIBA	Alpha/beta fold hydrolase	0.72	2.182 × 10^–7^
Q5DL40_ACIBA	Iron (III) ABC transporter, ATP-binding protein	0.73	1.896 × 10^–7^
A0A5N0G0P4_ACIBA	Peptide synthetase	0.76	5.495 × 10^–7^
V5VEQ8_ACIBA	1-pyrroline-4-hydroxy-2-carboxylate deaminase	0.84	4.602 × 10^–6^
A0A090B5V5_ACIBA	Glutamyl-tRNA reductase (GluTR)	0.81	2.415 × 10^–6^
A0A059ZR51_ACIBA	Protein adenylyl transferase	0.70	1.333 × 10^–7^
V5V8J8_ACIBA	Probable potassium transport system protein kup	0.71	1.584 × 10^–7^

### Lithium complex causes bacterial membrane disruption and permeabilization

**Fig 5:**
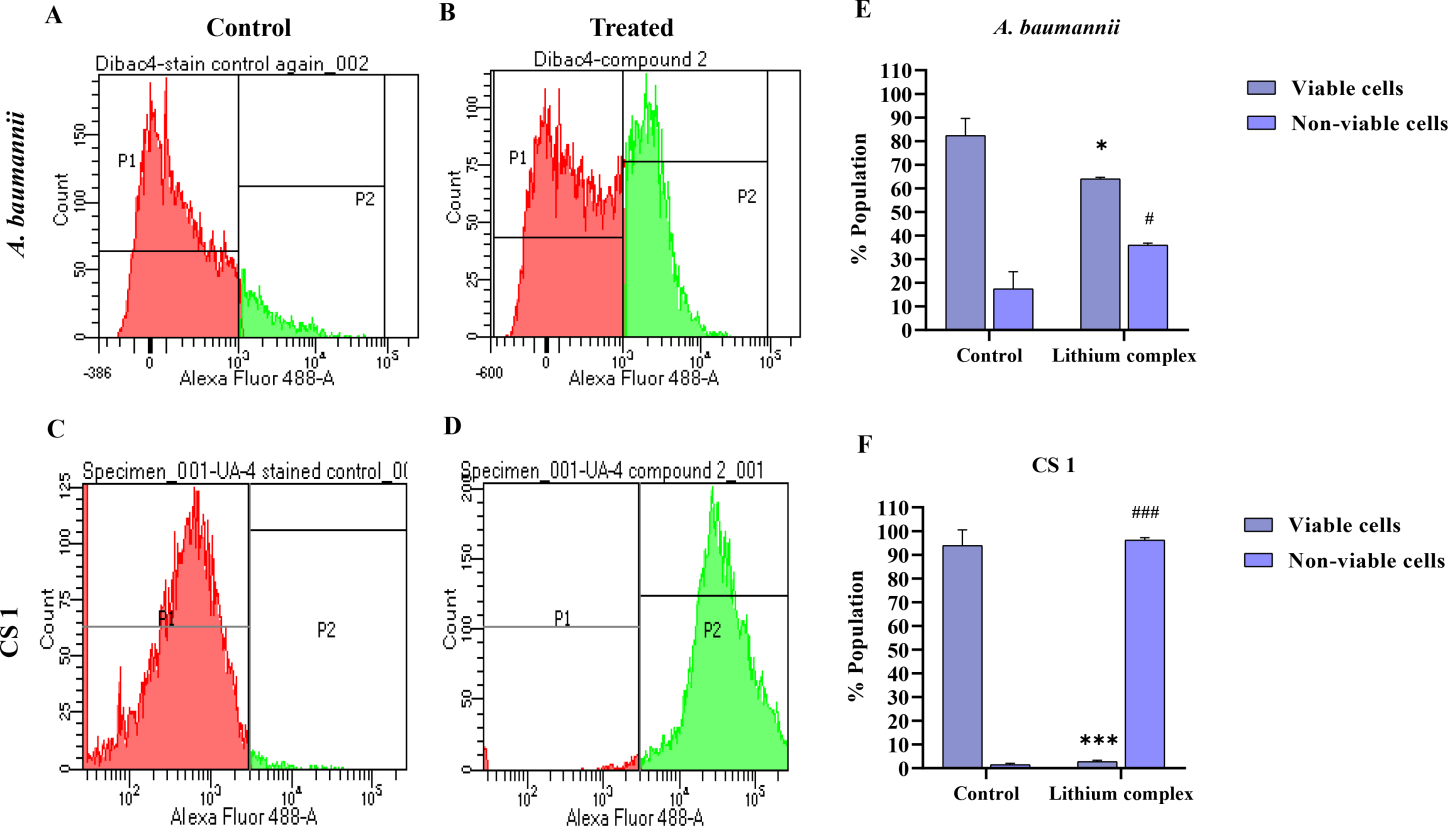
Flow cytometric analysis of membrane potential and integrity after treatment with lithium complex. (**A, C**) *A. baumannii* ATCC and CS 1 control. (**B, D**) *A. baumannii* ATCC and CS 1 treated with complex. (**E, F**) Graphical representation of treated and non-treated *A. baumannii* ATCC 19606 and CS 1. * represents comparison in viable cells while # represents non-viable cells before and after treatment with lithium complex. Statistical significance of data was represented as * and # at *P* < 0.05 and *** and ### at *P* < 0.001.

DiBAC_4_ was used to confirm the effect of lithium complex on membrane potential and structural integrity of *A. baumannii* ATCC and CS 1. Stain-labeled control of both strains revealed that >80% of the bacterial population was in region P1 which represent healthy and live cells (Fig. 5A and C). However, when *A. baumannii* ATCC strain was treated with lithium complex at MIC dose, there was a sudden and significant shift (30%) toward the P2 region which showed green fluorescence emitting depolarized cells that have lost their membrane potential and structural integrity (Fig. 5E). In case of CS 1, >90% of the population shifted significantly toward the P2 region with increase in green fluorescence intensity and implying majority of the bacterial cells were depolarized in cell population (Fig. 5B, D, and F). It was hence established that lithium complex significantly disrupted the bacterial membrane in both *A. baumannii* strains to cause increased membrane permeability and cellular rupturing.

### *In vivo* assessment of lithium complex in mice pneumonia model

**Fig 6:**
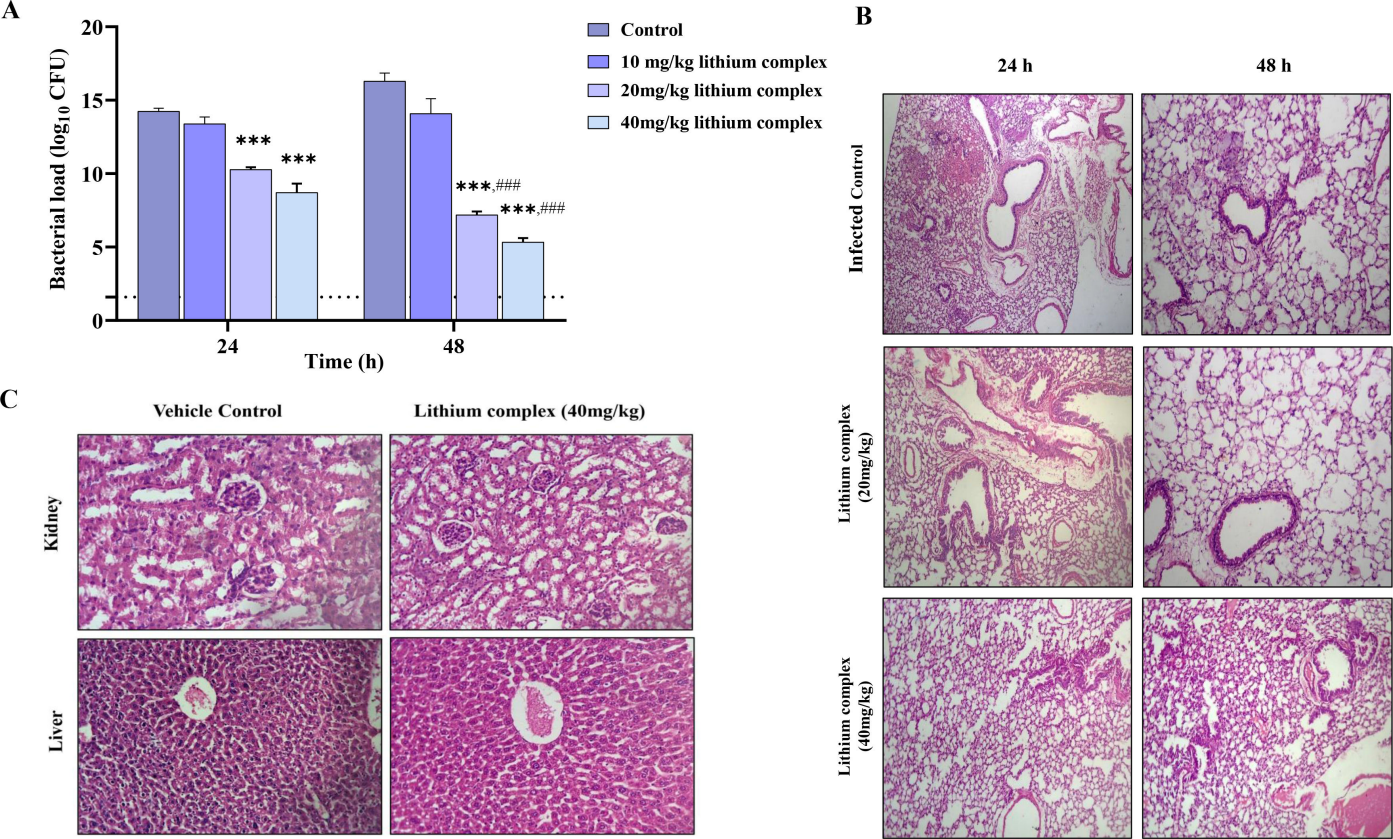
*In vivo* efficacy of lithium complex in *A. baumannii*-induced pneumonia. (**A**) Graphical representation of bacterial burden of infected control and lithium complex-treated mice at 24 h and 48 h. (**B**) Pulmonary parenchyma sections of control and lithium complex-treated mice at 24 h and 48 h. Statistical significance of data was evaluated using two-way ANOVA followed by Sidak’s multiple comparison test such that *** and ### represent *P* < 0.001. * is significant comparison in control and lithium complex-treated groups, while # is comparison between two different time points (24 h and 48 h) of the same group. 1.6 log_10_ CFU is the limit of detection. (**C**) Liver and kidney sections of vehicle control (normal saline) and lithium complex-treated mice.

The lithium complex was tested for its *in vivo* efficacy in *Acinetobacter baumannii*-induced pneumonia model. Mice in infected control had clinical score of −2 with a bacterial load of 14.25 ± 0.19 log_10_ CFU at 24 h which increased to 16.30 ± 0.470 log_10_ CFU at 48 h (Fig. 6A). Moreover, histopathological analysis of parenchymal sections revealed severe scarring indicating necrosis accompanied by extreme inflammation and fibrosis inflicted by *A. baumannii* (Fig. 6B). This persistence of infection indicates the successful development of pneumonia without any significant bacterial clearance by mice immune system. Among the treatment groups, non-significant bacterial clearance was observed at 10 mg/kg of lithium complex. In contrast, mice treated with 20 mg/kg showed clinical score of 0 with significant reduction of bacterial load to 10.28 ± 0.12 log_10_ CFU at 24 h which further reduced to 7.20 ± 0.18 log_10_ CFU at 48 h. Histopathological images of mice treated with 20 mg/kg dose revealed lesser inflammation, fibrosis, and alveolar damage as compared to infected control. Furthermore, mice treated with 40 mg/kg lithium complex also had no clinical symptoms of pneumonia and showed bacteriological reduction to 8.72 ± 0.51 log_10_ CFU at 24 h which further reduced to 5.35 ± 0.22 log_10_ CFU at 48 h. The lung sections of this treatment group had notable decrease in alveolar distortion and edema in pulmonary parenchyma at 24 h and 48 h. There were no signs of hepatoxicity and nephrotoxicity at 40 mg/kg of the compound as shown in (Fig. 6C). These findings show the lithium complex antimicrobial and anti-inflammatory potential against pneumonia infection in mice without toxicity.

## DISCUSSION

Multi-drug-resistant *Acinetobacter baumannii* has emerged as a substantial threat worldwide with severe clinical manifestations like ventilator-associated pneumonia and bloodstream infections leading to escalating morbidity and mortality rates particularly in hospital settings (42). In this study, three of the clinical *A. baumannii* strains were found to be resistant to aminoglycosides, cephalosporin, carbapenems, and last resort drug colistin. Due to this ever-growing multi-drug resistance, organic compounds and their derivatives have been a major focus of drug research in contrast to metals, due to their potential toxicity. However, vigorous screening had indicated that metallodrugs are 10 times more antimicrobial than organic compounds (21, 43). Metallodrugs of transition metals have been extensively studied but there are insufficient data to decipher the untapped potential of alkali metal complexes, especially against *Acinetobacter baumannii* (44). So, this study examined a lithium complex based on 1,10-phenanthroline (phen) and salicylic acid (non-steroidal anti-inflammatory drug) for antimicrobial potential against clinically isolated multi-drug-resistant *A. baumannii* (28).

The antibacterial activity of lithium complex against these strains showed that it inhibits bacteria at MIC_90_ 16 µg/mL against ATCC and CS 1 of *A. baumannii* and at MIC_90_ 32 µg/mL for the other two CS. It is lesser than the reported MIC of salicylic acid alone, i.e., 500 µg/mL against Gram-negative and -positive pathogens (*Escherichia coli, Staphylococcus aureus, Pseudomonas aeruginosa, Enterococcus faecalis*), indicating its higher antimicrobial potential at lower dosage. This can be due to the Phen ring which is reported to play a role in increasing potency against various Gram-positive and Gram-negative microbes (45, 46). In a recent study, the antibacterial activity of a metal salt (lanthanum salt) was compared to its complex (phen {La (III) complex}) and it was observed that the complex was more effective against *Escherichia coli and Staphylococcus aureus* (47). Growth kinetics is an important pharmacodynamic aspect which was used to assess bacterial inhibition at various time points (48). The lithium complex began to demonstrate its inhibitory action after 2–3 h against all the strains which showed that this compound can delay bacterial growth from 2 to 24 h. 2× MIC and MIC dose showed similar bacteriostatic effect against all *A. baumannii* strains throughout the experiment.

Biofilm is a protective shield that plays a major role in pathogenicity of MDRAB, making them impermeable to antimicrobial drugs and harsh chemicals. It also enables them to cross-exchange antibacterial resistance genes among various species within the biofilm (49). In this study, ATCC strain and clinical isolates of *A. baumannii* produced notably strong biofilm with OD_590nm_ >2.0. Previously, it was reported that phen, when used alone for biofilm-inhibiting potential, reduced ∼23% of biomass. However, when it was incorporated with metals such as silver (Ag) and copper (Cu), a significant increase in biofilm inhibitory potential of ∼48% was observed against *Pseudomonas aeruginosa and* its clinical isolates (50). Therefore, when the lithium complex was analyzed for its biofilm inhibition, it significantly inhibited 80%–85% of biofilm against *A. baumannii* and its clinical strain. Besides this, it significantly interrupted the formation of biofilm at MIC level against all the respective strains, with >85% biofilm inhibition. Mostly pathogenic bacteria develop mature biofilms on hospital surfaces and medical devices, which are difficult to eradicate due to their high tolerability against antimicrobial drugs (51). As most of the currently available treatments are rendered ineffective due to the inability of breaching this biofilm barrier, lithium complex was assessed for biofilm eradication and viability inhibition potential. The lithium complex significantly dispersed >70% of pre-formed mature biofilm of all strains of *A. baumannii* at MIC. Moreover, it was observed that the compound significantly reduced >75% of biofilm viability of both strains of *A. baumannii* at MIC level. It is an indication that the metal complex can penetrate the biofilm and has powerful anti-adherent activity to exhibit strong anti-biofilm property against *A. baumannii* strains. LM and atomic force microscopy further confirmed the biofilm dispersal activity of lithium complex. At MIC, the pre-formed mature biofilm after treatment exhibited disarticulation of biofilm network with detachment and void spaces in comparison to dense, thick, and compact mass of biofilm in all respective strains of *A. baumannii*. Also, AFM results revealed that after exposure of biofilm with lithium complex at MIC, the 3D configuration network of biofilm was distorted with damage of extracellular polymeric substance (EPS). Additionally, bacterial cells within biofilm lost structural integrity in comparison to untreated biofilm which shows healthy morphology of cells of *A. baumannii*.

The mechanism of action of the lithium complex was explored through proteomic analysis. It was observed that universal stress proteins, catalases, and peroxidases were highly regulated, which is an indicator of oxidative stress inflicted on *A. baumannii* (52). ROS accumulation including H_2_O_2_ (hydrogen peroxide), ^1^O_2_ (singlet oxygen), OH- (hydroxyl radical), and O_2^−^_ (superoxide anion) had previously been reported as a source of cell death through metal complexes action in bacterial pathogens (53, 54). This result shows upregulation of elongation factors and overall proteins of translation which is in coherence with the previous studies suggesting oxidative stress has a strong effect on central dogma, and biochemical processes are regulated accordingly to prevent cellular damage (55). Downregulation of various enzymes like peptide synthase, 1-pyrroline-4-hydroxy-2-carboxylate deaminase, alpha/beta hydrolase, and glutamyl tRNA reductase was also observed, implying the disruption in biosynthesis of various macromolecules. Consequently, downregulation of outer membrane proteins and cell envelope integrity proteins might have occurred to contribute to cell rupturing that resulted in leakage of intracellular proteins. These results also demonstrate lithium complex as an anti-virulence compound as it caused reduced expression of AraC transcriptional regulators, outer membrane protein (OmpA), KUP potassium transporter, and iron (III) ABC transporter. These virulence factors play a major role in adherence, persistence, and survival of *A. baumannii* during its pathogenesis (51, 56 – 58). Then, we further investigated the effect of the lithium complex on bacterial membrane by performing membrane potential disruption assay through a sensitive membrane potential dye DiBAC_4_. The results showed that lithium complex rapidly instigated the depolarization of *A. baumannii* ATCC 19606 and other clinical isolates to cause the death of cells confirming the rupturing of cell membrane and loss of cytoplasmic content.

Nevertheless, *A. baumannii* biofilms on medical devices have surged hospital-acquired pneumonia infection rates in ICUs as they can escape immune surveillance as well as any administered therapeutic treatments (59). Therefore, after observing the anti-virulence and anti-biofilm potential of lithium complex, it was further tested *in vivo* for its safety, efficacy, and potency at various concentrations against MDRAB-based pneumonia in mice. It was evaluated that the compound had dose-dependent efficacy with reduction in bacterial load at 20 and 40 mg/kg/day but found inactive at 10 mg/kg. It was observed that bacterial load at 20 mg/kg was 1.38 times lesser than infected control at 24 h which changed to a 2.26-fold reduction in 48 h. Similarly, mice treated with 40 mg/kg lithium complex showed higher efficacy with 1.63 and 3.04 times reduced bacterial load at 24 h and 48 h as compared to their respective controls. Pulmonary parenchyma of infected mice showed widespread bacterial colonization with congested and distorted alveolar spaces which indicates successful induction of infection as mentioned in previous studies (60). However, the lithium complex exhibited no toxicity up to 40 mg/kg and caused a reduction in bacterial load. Therefore, there was lesser alveolar damage, edema, and inflammation, highlighting its therapeutic potential against *A. baumannii*-induced pneumonia. Moreover, the lithium complex prevented further parenchymal deterioration and worsening of symptoms until 48 h, suggesting that continuity of regimen might have eradicated the infection completely. Hence, further inquiry alongside pharmacological aspects is required for better understanding of the potential of the compound. Consequently, according to our findings, the lithium complex has a strong antimicrobial potential both *in vitro* and *in vivo* against nosocomial infections causing MDRAB.

### Conclusion

We conclude that Li(phen)_2_sal has a strong potential to inhibit bacterial growth and reduce mature biofilm mass of MDR *A. baumannii*. This metal complex employs oxidative stress as one of its mechanisms to evade the biofilm barrier and have *in vitro* and *in vivo* efficacy against *A. baumannii*. Therefore, this study suggests that lithium complex has strong antimicrobial, anti-virulent, anti-adherent, and anti-biofilm activity that can be used in treating MDR nosocomial infections caused by *A. baumannii* in hospital and community setups.
